# Research trends of protein palmitoylation in cancer from 2004 to 2024: a bibliometric and visualization analysis

**DOI:** 10.3389/fonc.2025.1571870

**Published:** 2025-06-23

**Authors:** Yufeng Peng, Kewei Peng, Yi Wang, Luyao Li, Yuefei Lu

**Affiliations:** ^1^ Department of Oncology, Zhenhai Hospital of Traditional Chinese Medicine, Ningbo, China; ^2^ The Rehabilitation Department of Wuhan No. 4 Hospital, Wuhan, China

**Keywords:** visualization analysis, research frontiers, palmitoylation, cancer, bibliometrics

## Abstract

**Background:**

Protein palmitoylation is a dynamic and reversible lipid modification that has attracted increasing attention in cancer research in recent years. Palmitoylation involves the covalent attachment of palmitic acid (C16) to cysteine residues, altering the protein’s hydrophobicity and thereby affecting its membrane localization, stability, and functional activity. Recently, palmitoylation has been closely associated with the development and progression of various cancers, making it a key factor in cancer biology research.

**Methods:**

This study conducted a systematic bibliometric analysis using the Web of Science Core Collection (WoSCC) as the data source. A total of 685 papers published between January 1, 2004, and December 31, 2024, on the relationship between protein palmitoylation and cancer were selected. Information such as article titles, abstracts, and keywords was extracted to analyze publication trends, research hotspots, and collaboration networks among authors and institutions, thus assessing the dynamics of research in this field.

**Results:**

The analysis revealed that from 2004 to 2024, a total of 685 papers were published on the relationship between protein palmitoylation and cancer, with a significant increase in publications after 2020. The United States and China are the leading countries in this field, with institutions like Harvard University and the Chinese Academy of Sciences making substantial contributions. Research hotspots have shifted from early mechanistic studies to cancer-specific applications, particularly in areas such as tumor immune evasion, metabolic reprogramming, and therapeutic strategies, where significant progress has been made.

**Conclusion:**

Future efforts should concentrate on three primary directions: constructing high-resolution pan-cancer palmitoylation site maps to unveil subtype-specific modification patterns; developing subtype-selective inhibitors targeting the ZDHHC enzyme family to overcome the toxicity limitations of current broad-spectrum inhibitors; and establishing international research alliances to integrate China’s high productivity with the United States’ translational expertise, thereby bridging regional disparities between basic research and clinical innovation, ultimately advancing palmitoylation regulatory networks toward precision therapeutic strategies.

## 1 Introduction

Protein palmitoylation, a dynamic and reversible lipid modification, has gradually emerged as a crucial aspect of post-translational modifications, which themselves play an essential role in regulating protein function, cellular behavior, and intracellular interactions (1). Recent advancements in cancer research have significantly expanded our understanding of oncogenesis and therapeutic strategies, with palmitoylation increasingly recognized as a key factor influencing tumor biology (2). This modification, characterized by the covalent attachment of palmitic acid (C16) to cysteine residues, alters the hydrophobicity of proteins, thereby affecting their membrane localization, stability, and functional activity (3). Notably, palmitoylation is catalyzed by the zinc-finger DHHC (ZDHHC) family and reversed by depalmitoylating enzymes such as APT1 and APT2, highlighting its role as a molecular switch that modulates protein activity in various physiological and pathological contexts, including cancer progression (4).

Extensive research has linked palmitoylation to the regulation of oncogenes such as RAS, EGFR, and PD-L1, as well as tumor suppressors like SCRIB and PTEN, revealing its crucial role in oncogenic signaling pathways, metabolic reprogramming, and immune evasion mechanisms (5–7). For instance, studies have shown that ZDHHC20-mediated palmitoylation enhances MYC mRNA stability, thereby accelerating pancreatic cancer progression (8, 9), while palmitoylation of PCSK9 has been implicated in the activation of the PI3K/AKT pathway, contributing to sorafenib resistance in hepatocellular carcinoma, which is particularly significant given the ongoing efforts to develop effective targeted therapies (10). Furthermore, research has begun to uncover the impact of palmitoylation on immune evasion, with findings indicating that palmitoylation of IFNGR1 promotes its lysosomal degradation, thereby impairing the immune response against cancer cells, a discovery that underscores the potential of targeting palmitoylation in immuno-oncology (11, 12). Despite the significant progress in elucidating the molecular mechanisms and therapeutic implications of palmitoylation in cancer, substantial challenges remain, particularly in understanding the dynamic regulation of palmitoylation and its context-dependent functions across different cancer types, which necessitates further investigation (13). Another major obstacle lies in the development of palmitoylation-targeted therapies, as issues such as poor selectivity and low bioavailability continue to hinder the translation of these findings into clinical applications (14). Although palmitoylation has been shown to be a key player in various cancers, comprehensive studies analyzing its commonalities and specificities across different cancer types remain scarce, which in turn limits our ability to formulate generalizable therapeutic strategies (15, 16). Moreover, a systematic examination of the global research landscape in this field is still lacking, making it imperative to employ quantitative analytical methods to map out its development. Bibliometric analysis provides a powerful tool for quantitatively assessing research activities by examining factors such as publication trends, geographic distribution, collaboration networks, and emerging research hotspots, allowing us to gain a clearer picture of the trajectory of palmitoylation research within the realm of cancer biology. To this end, the present study applies bibliometric techniques to systematically analyze the literature on protein palmitoylation in cancer, leveraging comprehensive database resources to evaluate research trends, key focus areas, and future directions. By conducting an in-depth assessment of the global distribution of publications, thematic research areas, and patterns of academic collaboration, this study aims to offer novel insights into the mechanistic roles of palmitoylation in cancer as well as its potential applications in clinical oncology, thereby providing a valuable reference for future research efforts and therapeutic developments in this rapidly evolving field. This study aims to address the research hypothesis that regional dominance, research translation gaps, and thematic convergence in protein palmitoylation studies in cancer research reveal distinct global and temporal trends, with significant implications for therapeutic strategy development.

## 2 Materials and methods

### 2.1 Search strategy

The Web of Science Core Collection (WoSCC), globally recognized as an authoritative database, served as the primary source for this bibliometric study. We specifically utilized the Science Citation Index Expanded (SCIE) within the WoSCC to retrieve relevant literature on protein palmitoylation in cancer, published from January 1, 2004, to December 31, 2024.The retrieved dataset comprised comprehensive citation metadata essential for a robust knowledge graph analysis, including article titles, abstracts, keywords, author affiliations, institutional and country distributions, citation frequencies, and collaborative networks. To formulate a precise and comprehensive search strategy, we initially consulted the NIH National Library of Medicine (NLM) database. From this, we identified 17 Medical Subject Headings (MeSH) terms related to “Neoplasms” and 3 terms associated with “protein palmitoylation.” Based on these MeSH terms, relevant keywords, and the specific objectives of our study, we developed a detailed search query, which is provided in **Supplementary Data Sheet 1**.

**Figure 1:**
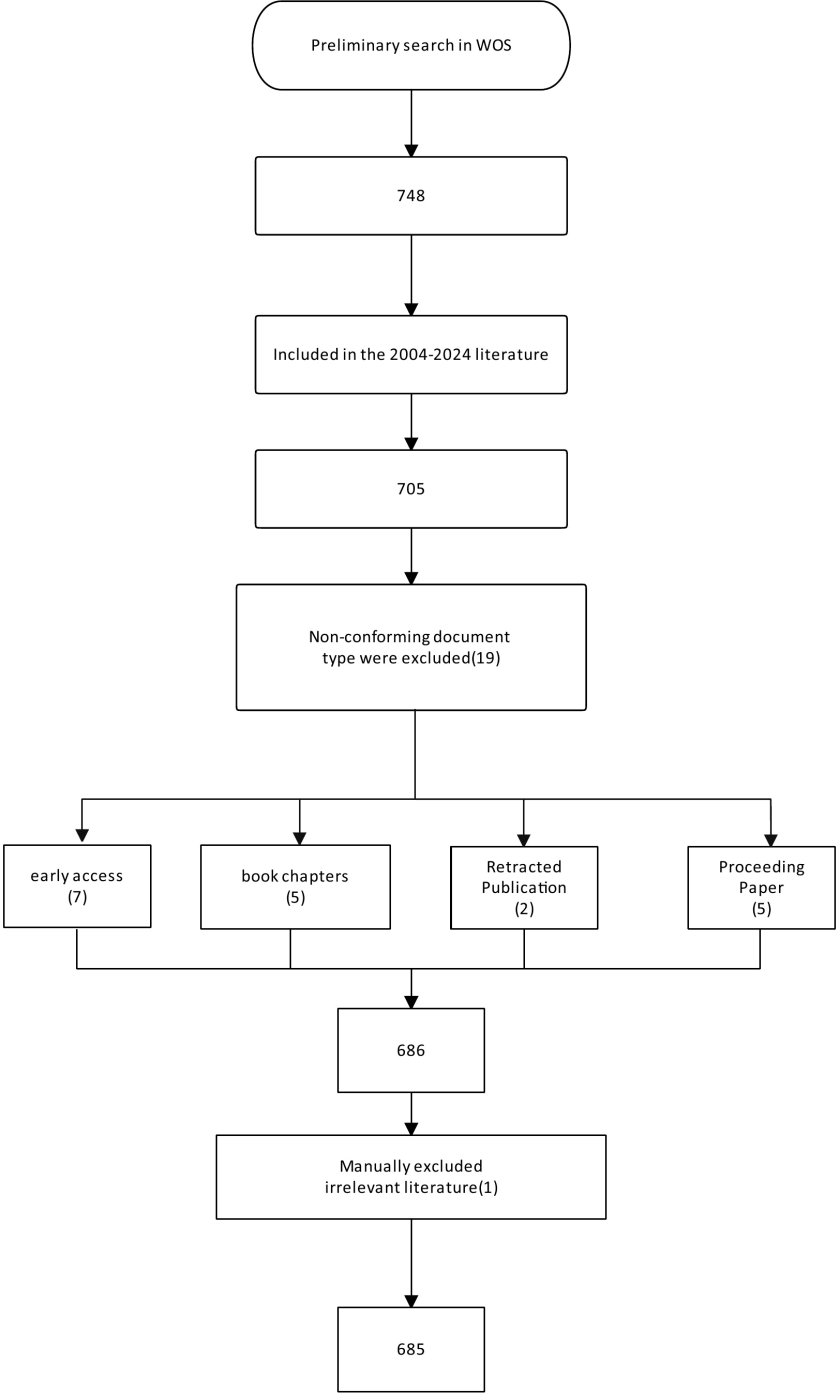
Flowchart of the study.

To ensure the relevance and quality of the included literature, document types were restricted to Articles and Review Articles. We systematically excluded editorial materials, conference abstracts, early access publications, notes, book chapters, letters, retracted publications, and corrections. Following an initial screening and abstract review, 68 irrelevant documents were excluded. The final dataset for analysis comprised 685 publications. The detailed flow of the literature selection process is illustrated in **Figure 1**.

### 2.2 Data processing and analysis

Following the retrieval and download of raw data from the WoSCC database, all titles and abstracts underwent independent screening by two reviewers (Yufeng Peng and Kewei Peng). The data were exported from the WoSCC database in plain text format, with each file uniquely named as “download_xxx.txt”. The exported data encompassed essential information including article titles, publication years, authors, research institutions, keywords, abstracts, and journal details. For data analysis and visualization, a suite of specialized software tools was employed. Processon was utilized for creating the flowchart, while Origin 2024 was used to generate trend graphs. SCImago Graphica 1.0.48 facilitated the visualization of country collaboration networks. VOSviewer 1.6.2 was instrumental in mapping institutional collaboration networks and keyword co-occurrence. Bibliometrix 4.1.3 was used to construct author-related figures. Finally, CiteSpace 6.4 R1 was used for the analysis and visualization of keyword bursts and co-cited references.

## 3 Result

### 3.1 Publication growth trend

**Figure 2:**
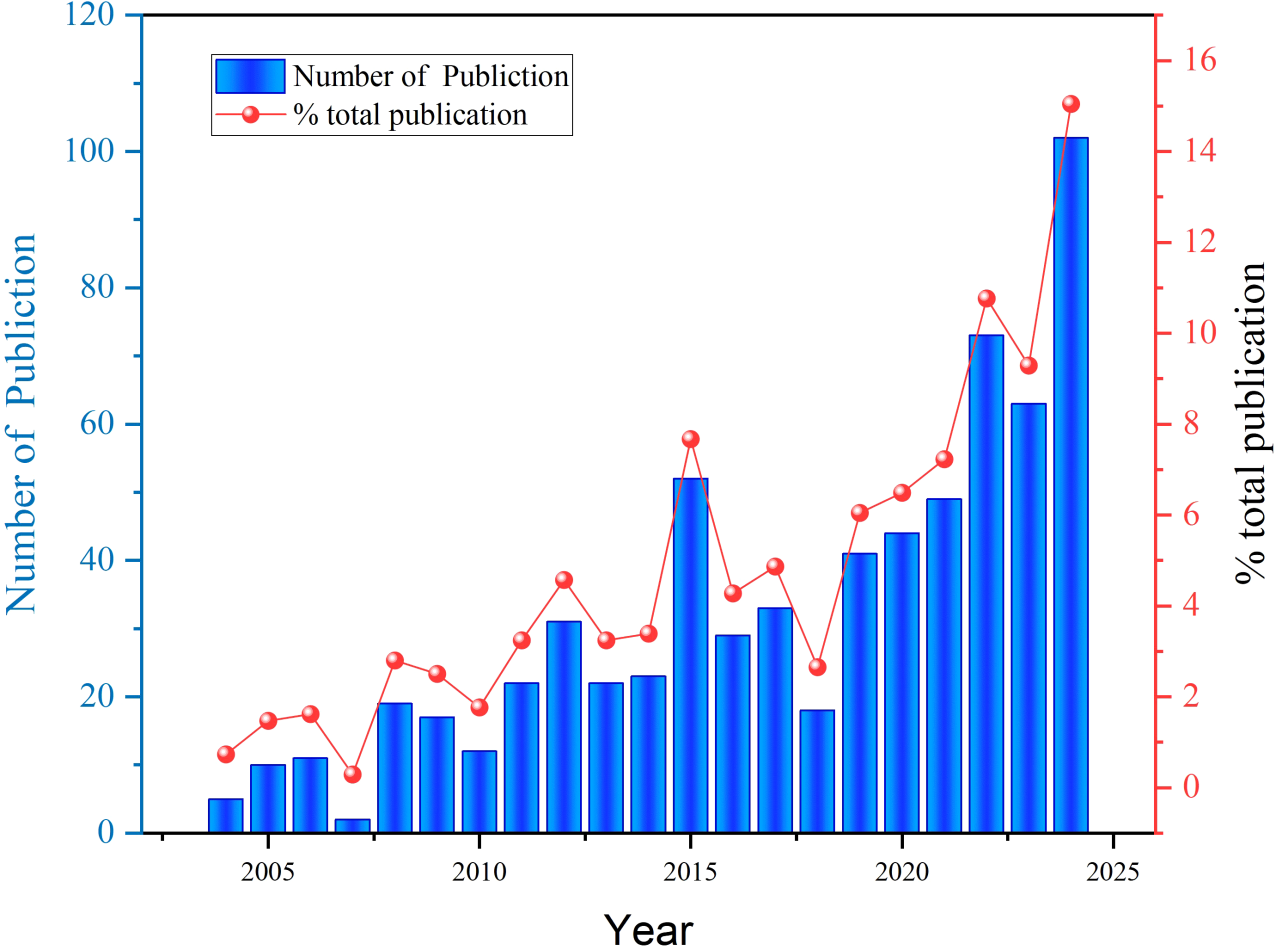
Trends in annual publications and cited articles from 2004 to 2024.

The growth trend of publications provides insights into the development and increasing prominence of protein palmitoylation research in the field of cancer. As depicted in **Figure 2, a** total of 685 publications were recorded between 2004 and 2024, illustrating the evolutionary trajectory of this research domain. In 2004, only five papers were published, indicating that the field was in its nascent stage. From 2004 to 2008, the growth remained sluggish, with the annual publication count fluctuating in single digits. However, by 2010, the number of publications rose to 13, marking the initial phase of heightened research interest. A significant surge was observed between 2010 and 2012, with publications increasing from 13 to 32, followed by steady expansion in 2014 (23 papers) and 2016 (52 papers), signaling a period of accelerated academic engagement. The notable increase during this phase suggests that an expanding number of researchers contributed to this field, leading to a substantial body of accumulated findings. Nevertheless, after reaching a local peak in 2016 (52 papers), the publication rate declined, hitting its lowest point in 2018, when only 18 studies were published. Despite this downturn, research output rebounded in 2020, with the annual count rising to 67 papers, indicating a revival of interest in the field. From 2020 onward, the publication rate exhibited a consistent upward trend, remaining stable at 67 papers before experiencing a steady increase, ultimately reaching 105 publications in 2024, marking the highest annual output to date. This sustained growth underscores the emerging significance of palmitoylation in oncological research, reflecting its status as a critical area of scientific inquiry and therapeutic exploration.

### 3.2 Country/region and institutional distribution

**Figure 3:**
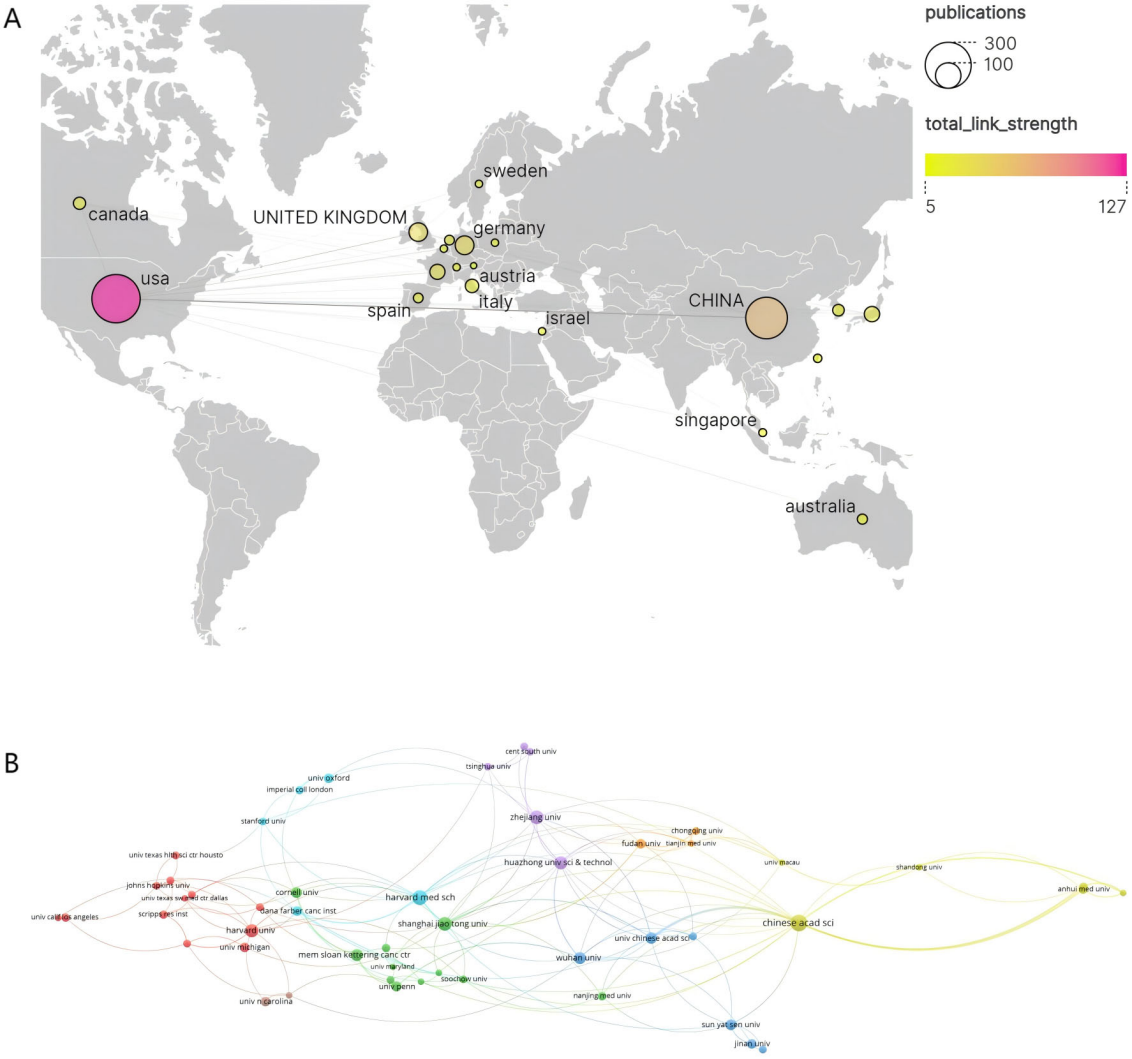
**(A)** Geographic visualization of country/area collaboration. **(B)** Co-analysis of the top 50 most productive institutions in the network visualization map.

The 685 publications in this study involved contributions from 883 institutions across 44 countries/regions. As shown in **Table 1** and **Figure 3A**, the top 10 most productive countries are ranked by publication volume. The United States (USA) holds an undeniable leadership position in this field, with significantly superior publication output (296 papers), total citations (17,316), average citations per article (58.50), and betweenness centrality(BC) (0.55). This not only indicates a vast scale of research output and strong influence but also a pivotal role as a core hub within the international collaboration network. China ranks second with 226 publications and, with a high total citation count (5,193) and BC (0.36) that is second only to the USA, stands as a crucial research contributor and collaborative node. Although Germany is third, its output of only 45 publications, along with relatively lower average citations per article (27.29) and centrality (0.16), suggests room for improvement in the average international impact of its research and its core role in collaborative networks. Notably, some countries, despite not having the highest total publication volume, exhibit significant research influence. Italy (average citations: 79.26), Canada (39.63), France (38.83), and Japan (34.70) are among the top in average citations per article, reflecting the high quality and impact of their research outputs. Among these, Australia (0.3) and England (0.2) also demonstrate strong BC. In contrast, South Korea, while making it into the top ten for output, lags in all citation metrics and has a centrality of 0. Overall, the global landscape of protein palmitoylation research in cancer is dominated by the United States, followed by China, with several European countries and Australia making significant high-quality research contributions. Network analysis further confirms that the USA and China are key centers for international collaboration in this domain.

**Table 1:** Publications and citations in the top ten most productive countries/regions and institutions.

Country	Count	Citations	Average citation	Centrality	Institutions	Count	Citations	Average citation	Centrality
USA	296	17316	58.50	0.55	Harvard University	61	3210	52.62	0.43
China	226	5193	22.98	0.36	Chinese Academy of Sciences	32	986	30.81	0.07
Germany	45	1228	27.29	0.16	University of Texas System	24	2091	87.13	0.13
England	36	1206	33.50	0.2	Shanghai Jiao Tong University	18	700	38.89	0.04
Japan	30	1041	34.70	0.03	Zhejiang University	17	778	45.76	0.06
France	29	1126	38.83	0.03	Huazhong University of Science & Technology	16	606	37.88	0.03
Italy	23	1823	79.26	0.02	Memorial Sloan Kettering Cancer Center	16	783	48.94	0.02
Canada	19	753	39.63	0.03	Wuhan University	15	136	9.07	0.02
South Korea	17	395	23.24	0	Cornell University	14	472	33.71	0.02
Australia	13	365	28.08	0.3	Sun Yat-sen University	12	334	27.83	0.03

At the institutional level, Harvard University (61 papers), the Chinese Academy of Sciences (32 papers), the University of Texas System (24 papers), and Shanghai Jiao Tong University (18 papers) are the core drivers of research output in this field. In terms of research impact, Harvard University stands out with the highest total citations (3210), the highest BC (0.43), and an exceptionally high average citation rate (52.62 citations per article). The University of Texas System in the USA also performs well, with an average of approximately 87.13 citations per article and a centrality of 0.13. These institutions collectively represent high-impact research in the field. This pattern is visually represented in **Figure 3B**. The map illustrates a complex global network of institutional relationships, where connections between nodes indicate collaborative links. Institutions in the map are grouped into multiple clusters by different colors, clearly showing that collaboration is not evenly distributed but forms cooperative groups centered around specific institutions or regions. This aligns with the characteristics revealed in**Table 1**, highlighting the dominance of North American and East Asian institutions. Although most top institutions have generally low BC scores, implying that overall collaboration might be relatively dispersed or feature multiple centers, the map visually confirms the existence of close collaborative relationships, particularly within specific clusters. In summary, research on protein palmitoylation in cancer is primarily led by top universities and healthcare systems in the United States and China. These institutions not only contribute a substantial volume of high-impact literature but also form collaborative networks that powerfully drive knowledge production and exchange in the field, clearly delineating a global collaboration landscape centered around North America and East Asia.

### 3.3 Author co-occurrence analysis

**Figure 4:**
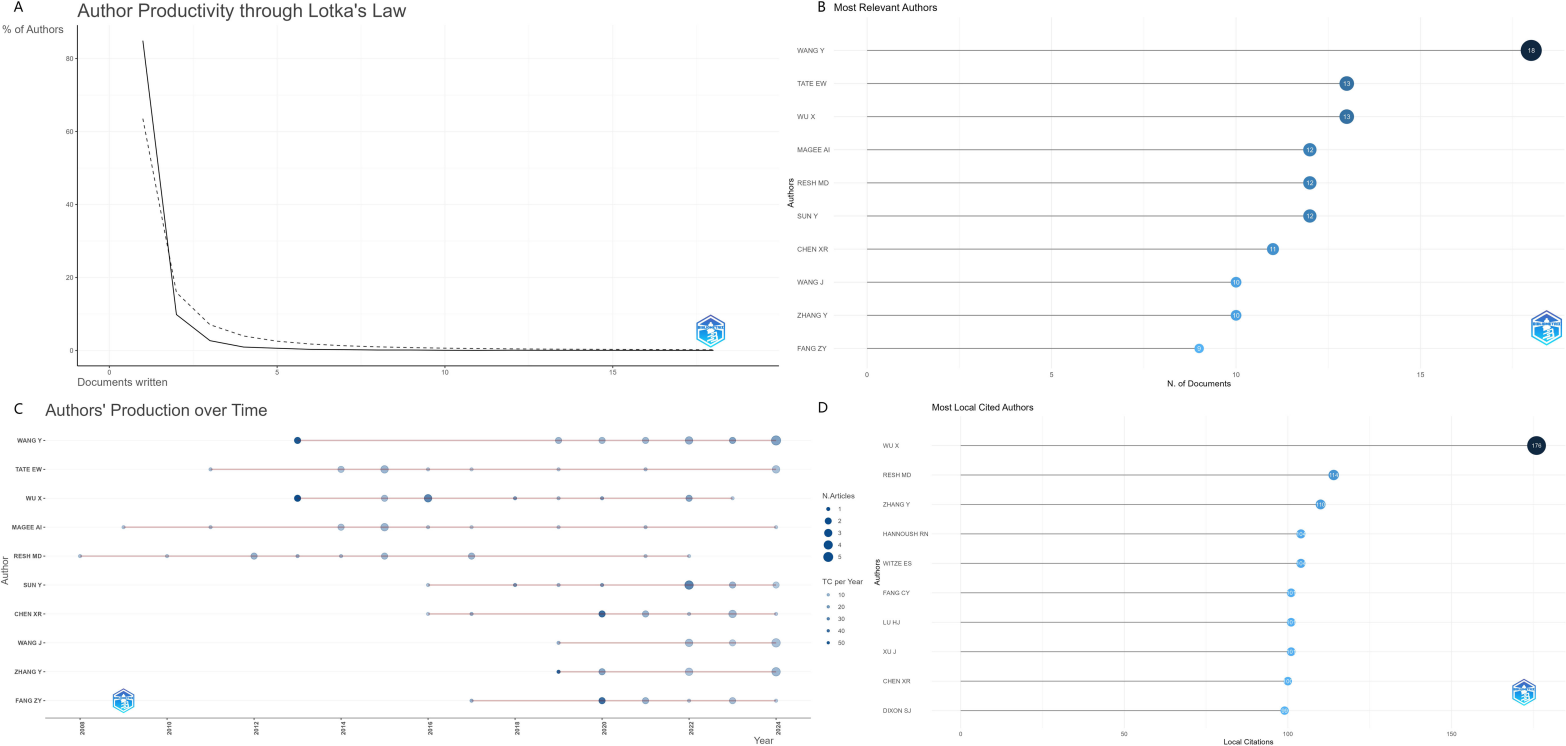
**(A)**Author Productivity through Lotka’s Law **(B)**Most Relevant Authors **(C)**Authors’ Production over Time **(D)**Most Local Cited Authors.

The co-occurrence analysis of authors provides valuable insights into key academic leaders, collaboration networks, and the extent of scholarly contributions in the field of protein palmitoylation in cancer research. A total of 3,347 authors have participated in this research domain, reflecting a diverse and expanding scientific community. Based on Lotka’s Law of scientific productivity (**Figure 4A**), the majority of authors (84.9%) have published only one paper, indicating a high proportion of occasional contributors. Meanwhile, 9.8% of authors have published two papers, while only 5.3% of authors have contributed three or more publications, demonstrating that a small core group of researchers plays a leading role in driving advancements in this field.

In terms of individual productivity, the most prolific author is Wang Y, with 18 publications (**Figures 4B, C**), followed by TATE EW (13 papers) and WU X (13 papers). However, when considering citation impact, the most highly cited authors (**Figure 4D**) are WU X (176 citations), RESH MD (114 citations), and ZHANG Y (110 citations), signifying their influence in shaping the scientific discourse on palmitoylation-related cancer research. Additionally, the H-index values for these key authors indicate consistent and impactful contributions: Wang Y (H-index = 13), WU X (H-index = 12), and RESH MD (H-index = 11). Their high citation counts and strong academic influence confirm that they are leading contributors to the advancement of protein palmitoylation research in cancer biology, playing pivotal roles in defining key mechanistic insights and potential therapeutic applications.

### 3.4 Journals and cited academic journals

There were 308 academic journals actively engaged in research on protein palmitoylation in cancer. **Table 2** provides a detailed list of the top 10 journals ranked by publication volume within this research area, including their 2023 Journal Citation Reports (JCR) data and Impact Factors (IF) as reported by Web of Science. The IF serves as a key metric for evaluating a journal’s quality, importance, and influence. In terms of journal output, the Journal of Biological Chemistry stands out, not only publishing the most relevant articles (N=30) but also accumulating the highest number of citations (1171). Its Impact Factor of 4 and JCR Q2 quartile status solidify its central role as a premier publication in this field, underscoring its significance as a platform for molecular mechanism research. Following closely are PLoS One (N=21, Citations=627, IF=2.9, JCR Q1) and Oncogene (N=13, Citations=667, IF=6.9, JCR Q1), indicating the growing role of open-access journals in disseminating palmitoylation research. Nature Communications and Scientific Reports also hold important positions in publishing relevant literature, highlighting the influence of high-impact multidisciplinary journals in spreading interdisciplinary palmitoylation research.

**Table 2:** Top 10 journals in terms of the number of publications and co-citation frequency.

Journal	Documents	Citations	JCR	Impact factor (2024)	Co-cited journal	Citations	JCR	Impact factor (2024)
Journal of Biological Chemistry	30	1171	2	4	Journal of Biological Chemistry	2819	2	4
PLOS ONE	21	627	1	2.9	PNAS	1395	1	9.4
Oncogene	13	667	1	6.9	Nature	1269	1	50.5
PNAS	12	1277	1	9.4	Cell	1092	1	45.5
Cancer Research	11	700	1	12.5	Cancer Research	968	1	12.5
Nature Communications	11	817	1	14.7	Science	790	1	47.7
Scientific Reports	11	104	2	3.8	Nature Communications	684	1	14.7
FASEB Journal	8	462	2	4.4	Journal of Cell Biology	664	1	7.4
Oncotarget	8	266	none	none	Oncogene	642	1	6.9
​​Biochemical and Biophysical Research Communications​	7	121	3	2.5	Nature Chemical Biology	510	1	12.9

An analysis of the most frequently co-cited academic journals reveals that the Journal of Biological Chemistry is the most co-cited with 2819 occurrences. This underscores its pivotal importance in the study of palmitoylation’s molecular mechanisms, signaling pathways, and protein functions. Proceedings of the National Academy of Sciences and Nature rank second and third with 1395 and 1269 co-citations, respectively, demonstrating the broad impact of palmitoylation research across diverse biological and medical fields. The high co-citation rates of Cell (Citations=1092) and Cancer Research (Citations=968) further emphasize the significance of palmitoylation in cancer biology, particularly in studies concerning cellular signaling, tumor proliferation, and metastasis. Science and Nature Communications also occupy prominent positions among co-cited journals, suggesting that research findings are not only widely embraced by top-tier basic science journals but also receive considerable attention in broad multidisciplinary publications.

### 3.5 Reference co-citation, clustering, timelines, and bursts

The most cited publications in the field of protein palmitoylation in cancer provide critical insights into key scientific advancements and emerging research directions. As outlined in **Table 3**, the most highly cited study, conducted by Han Yao et al., titled “Inhibiting PD-L1 palmitoylation enhances T-cell immune responses against tumors” (17), has been cited 79 times, highlighting its significant impact on tumor immunology and therapeutic strategies. This study demonstrated that palmitoylation of PD-L1 stabilizes its expression by preventing ubiquitination and lysosomal degradation, thereby enhancing its immune-suppressive function in tumor cells. By inhibiting PD-L1 palmitoylation using small-molecule inhibitors (such as 2-BP) or peptide-based approaches (CPP-S1), researchers successfully reduced PD-L1 expression and restored T-cell-mediated anti-tumor immunity, providing a strong theoretical foundation for the development of novel cancer immunotherapies, particularly as a complementary strategy to current antibody-based treatments.

**Table 3:** Protein palmitoylation was one of the top 10 most cited papers in cancer.

Co-cited reference	DOI	Citations	Journal	First author
Inhibiting PD-L1 palmitoylation enhances T-cell immune responses against tumours	10.1038/s41551-019-0375-6	79	Nat Biomed Eng	Han Yao
Protein palmitoylation and cancer	10.15252/embr.201846666	64	EMBO Rep	Pin-Joe Ko
Palmitoylation stabilizes PD-L1 to promote breast tumor growth	10.1038/s41422-018-0124-5	56	Cell Res	Yi Yang
DHHC9-mediated GLUT1 S-palmitoylation promotes glioblastoma glycolysis and tumorigenesis	10.1038/s41467-021-26180-4	35	Nat Commun	Zhenxing Zhang
A STAT3 palmitoylation cycle promotes TH17 differentiation and colitis	10.1038/s41586-020-2799-2	29	Nature	Mingming Zhang
Inhibition of DHHC20-Mediated EGFR Palmitoylation Creates a Dependence on EGFR Signaling	10.1016/j.molcel.2016.04.003	28	Mol Cell	Kristin B Runkle
Small Molecule Inhibitors of TEAD Auto-palmitoylation Selectively Inhibit Proliferation and Tumor Growth of NF2-deficient Mesothelioma	10.1158/1535-7163.MCT-20-0717	28	Mol Cancer Ther	Tracy T Tang
Protein Lipidation: Occurrence, Mechanisms, Biological Functions, and Enabling Technologies	10.1021/acs.chemrev.6b00750	26	Chem Rev	Hong Jiang
Palmitoylation: policing protein stability and traffic	10.1038/nrm2084	25	Nat Rev Mol Cell Biol	Maurine E Linder
Small-Molecule Covalent Modification of Conserved Cysteine Leads to Allosteric Inhibition of the TEAD·Yap Protein-Protein Interaction	10.1016/j.chembiol.2018.11.010	24	Cell Chem Biol	Khuchtumur Bum-Erdene

**Figure 5:**
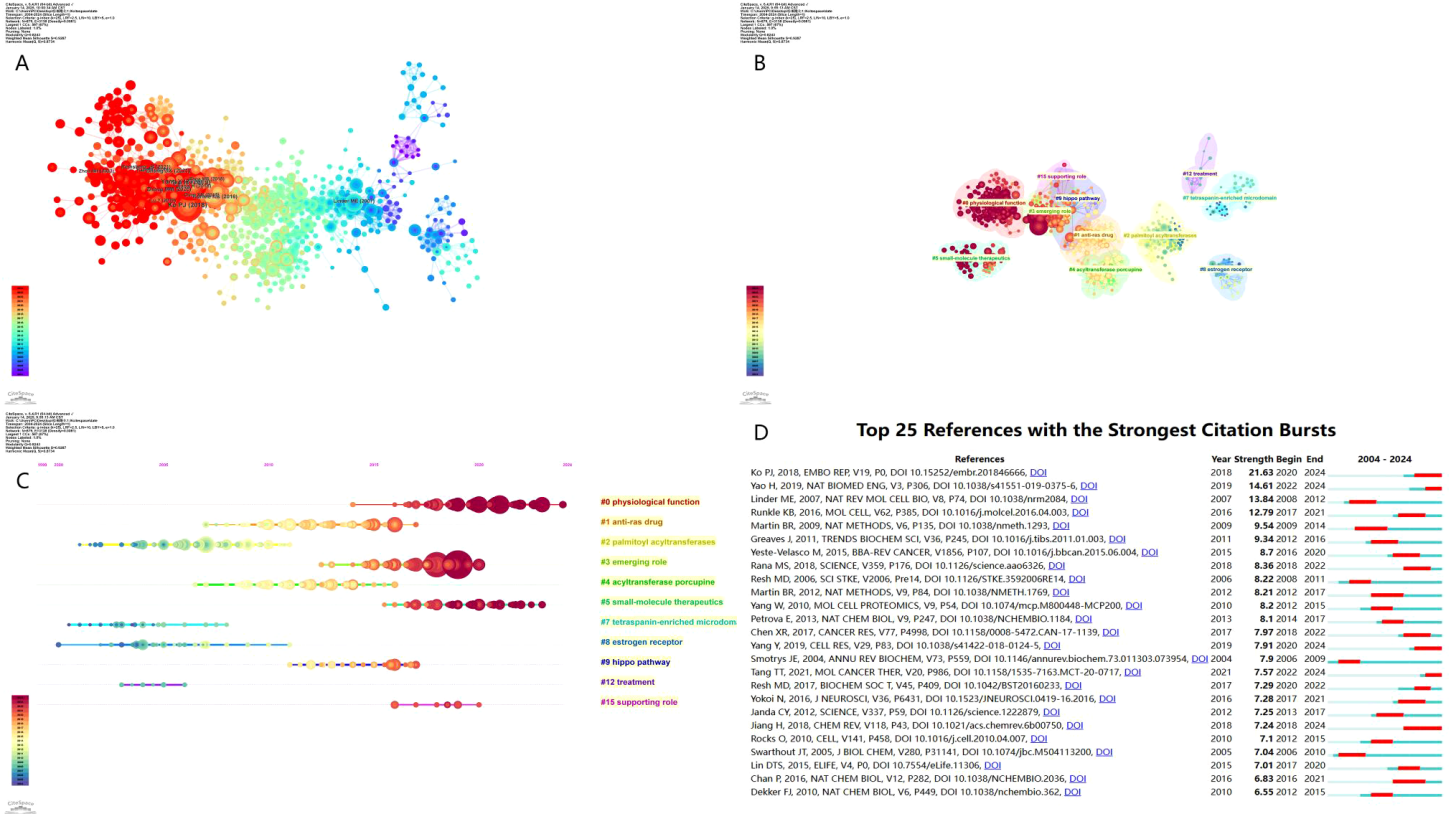
**(A)** Visualization of literature co-citations **(B)** Literature co-citations clustering **(C)** Timeline plot of literature co-citations clustering **(D)** The top 25 references with the strongest citation bursts.

The co-citation analysis of highly referenced studies (**Figure 5A**) reveals the interconnected nature of palmitoylation research, where different publications contribute to a knowledge network through citation relationships. In this network, node size represents citation frequency, while color indicates publication timeline. The red clusters signify foundational research that has had an early and significant impact, whereas blue clusters indicate more recent key studies. Notably, Ko PJ (2018) (1) and Linder ME (2007) (18) are positioned as core references, acting as key knowledge hubs in the field.

The thematic clustering of co-cited references (**Figure 5B**) categorizes the field into 16 research clusters, representing various stages of palmitoylation-related research. Key thematic groups include “Physiological Function”, which covers fundamental studies on the role of palmitoylation in normal cellular functions, serving as the foundation of the field; “Emerging Role”, which explores the expanding relevance of palmitoylation in cancer treatment and other diseases; “Hippo Pathway”, which focuses on the Hippo-YAP/TAZ signaling cascade and its role in cancer development; and “Treatment”, which is dedicated to therapeutic applications, particularly the development of small-molecule inhibitors targeting palmitoylation. These clusters represent the evolution of research priorities, shifting from basic mechanistic studies to translational and therapeutic applications.

The temporal evolution of co-cited references (**Figure 5C**) further delineates how research trends have shifted over time. The timeline view organizes references along a left-to-right gradient, where older studies on the left represent classic foundational research, while newer studies on the right indicate emerging frontiers. Research from 2005–2015 primarily focused on the molecular mechanisms and functional roles of palmitoylation, including enzyme activity, protein stability, and intracellular signaling pathways. In contrast, research from 2016–2024 has increasingly emphasized cancer-related applications, particularly in signaling regulation, drug resistance, and immunotherapy.

The burst detection analysis of co-cited references (**Figure 5D**) identifies highly influential papers with a sudden surge in citations over a given period, providing a quantitative measure of impactful research trends. Among them, Ko PJ (2018) exhibits the highest burst intensity (21.63), dominating recent discussions from 2020–2024, with a focus on the functional role of palmitoylation in protein localization and tumor progression, establishing new potential targets for cancer therapy. Similarly, Yao H (2019) (17) and Runkle KB (2016) (19) have led advancements in cancer signaling and therapeutic strategies, with their citation peaks observed between 2022–2024, reflecting their increasing influence in the field. Meanwhile, Linder ME (2007) remains a cornerstone in early palmitoylation research, demonstrating how S-palmitoylation regulates protein stability and trafficking, which laid the theoretical foundation for subsequent translational studies. This publication had its highest impact during 2008–2012, serving as a reference point for later developments in cancer-related research.

Overall, the evolution of research hotspots in protein palmitoylation and cancer can be divided into two major phases: foundational research (2005–2015), which focused on enzymatic mechanisms and molecular functions, as exemplified by works such as Rocks O (2010) (20)and Martin BR (2009) (21), and the translational research phase (2016–2024), where studies such as Yang Y (2019) (22)and Tang T (2021) (23)have expanded the field toward cancer-specific applications, particularly in oncogenic signaling, therapeutic interventions, and clinical strategies. These findings underscore how protein palmitoylation research is rapidly transitioning from fundamental molecular studies to applied cancer therapeutics, with increasing interest in targeting palmitoylation for drug development, personalized medicine, and immune modulation strategies.

### 3.6 Keyword co-citation, clustering/timeline burst

**Figure 6:**
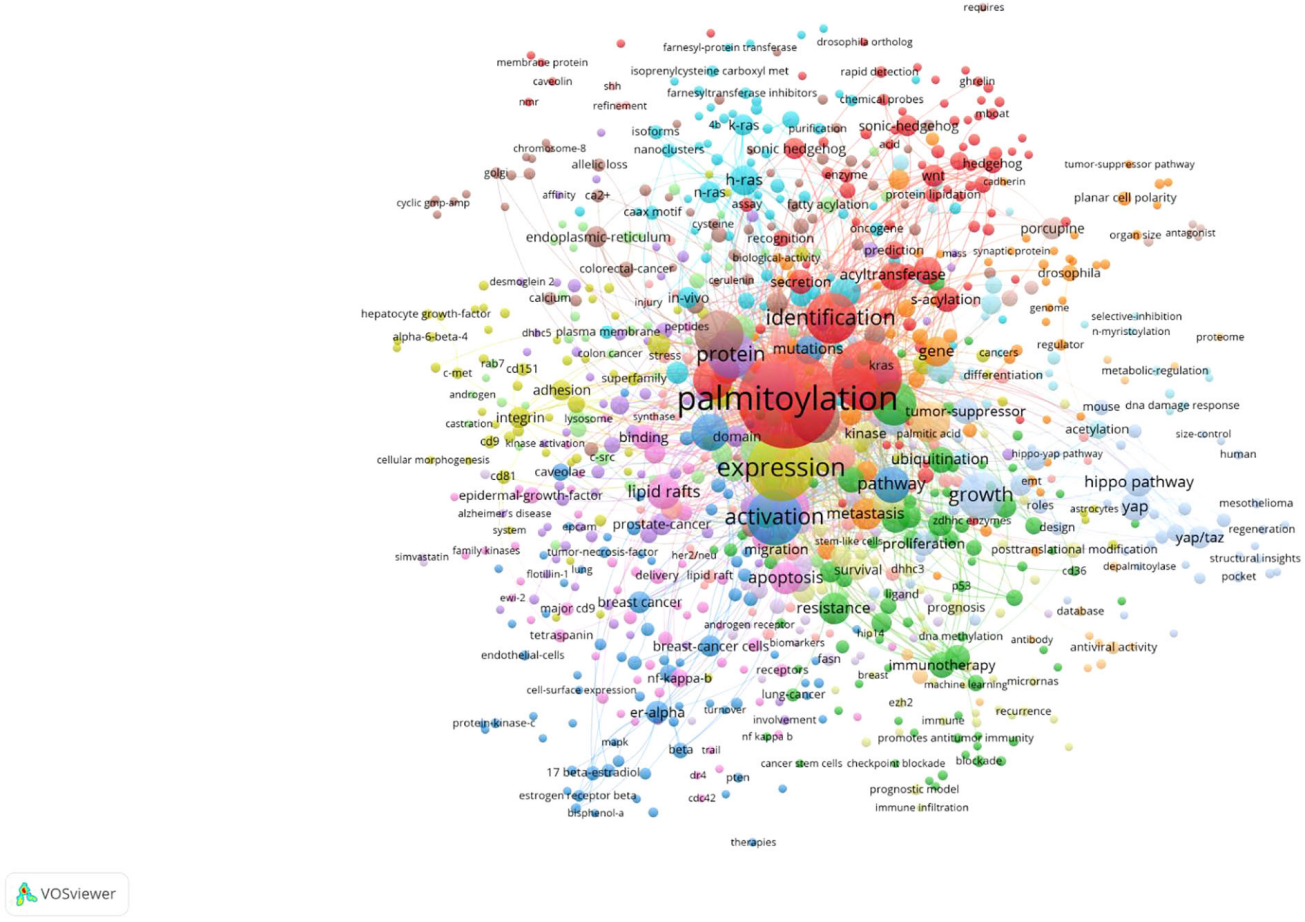
Network map of keywords.

**Figure 7:**
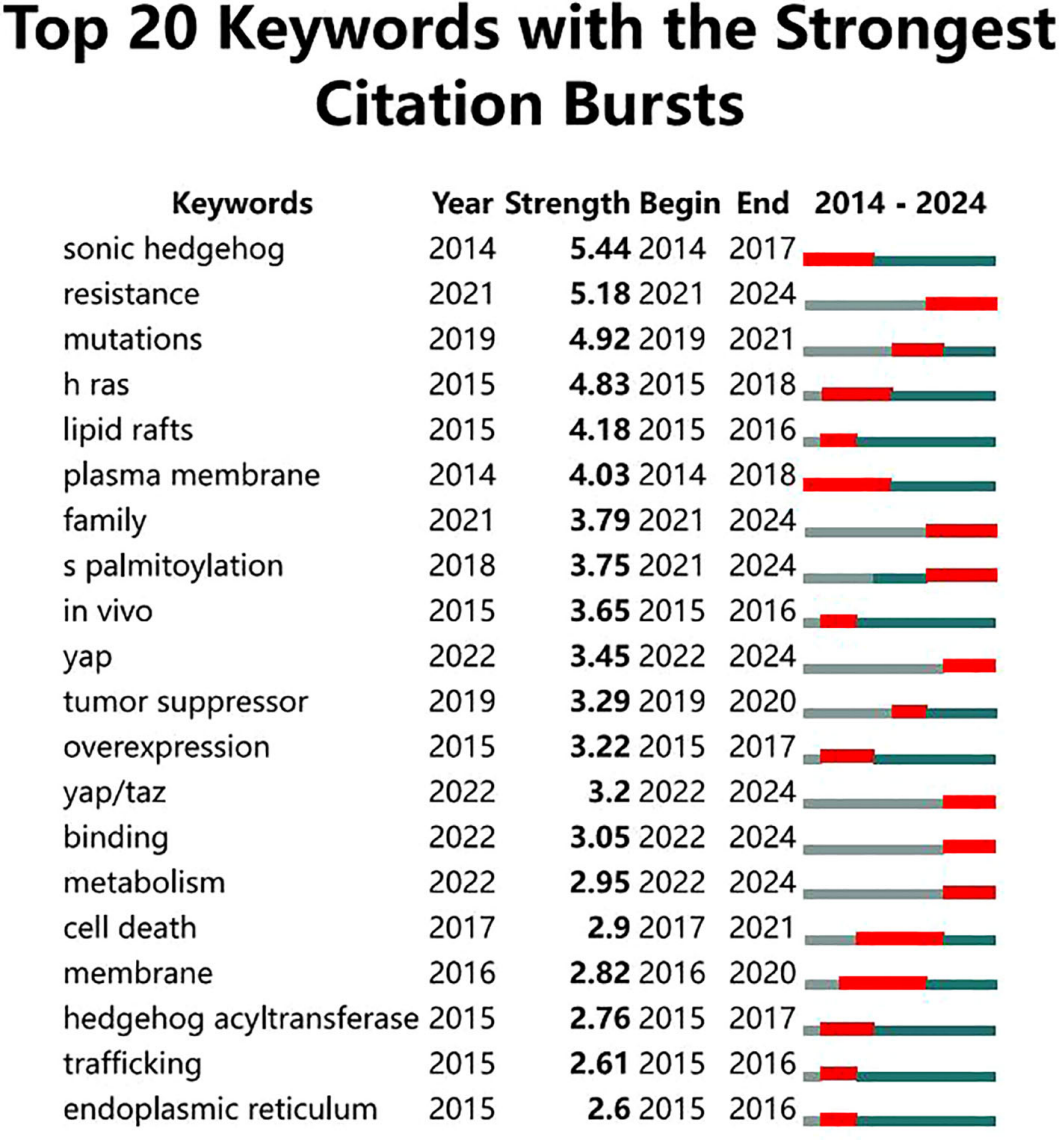
Top 20 keywords with the strongest citation bursts.

The keyword co-occurrence analysis (**Figure 6**) reveals major research themes and interconnections in protein palmitoylation studies, particularly in cancer biology. At its core, “palmitoylation” is closely associated with high-frequency keywords such as “expression,” “activation,” “pathway,” “growth,” and “mutations,” indicating its central role in protein regulation, cellular signaling, tumor progression, and mutation-driven oncogenesis. The keyword burst analysis (**Figure 7**) highlights research trends from 2004 to 2024, showing an evolution from molecular and structural investigations to cancer-specific applications. Early studies (2004–2016) were dominated by mechanistic research, as seen in keywords like “lipid rafts” (2004-2016) and “signal transduction” (2005-2009), reflecting foundational work on lipid signaling and protein-mediated cell communication, while “plasma membrane” (2005-2014) highlighted the role of palmitoylation in membrane protein localization and dynamics. In contrast, more recent research (2016-2024) has focused on cancer-related pathways and therapeutic interventions, with “S-palmitoylation” (2018-2024) underscoring increasing interest in its biochemical mechanisms, while “Hippo pathway” (2016-2024) and “YAP/TAZ” (2022-2024) indicate a growing focus on tumor proliferation and metastasis regulation via the Hippo-YAP/TAZ axis. Additionally, “therapy” (2021-2024) suggests that palmitoylation is being increasingly explored as a therapeutic target. This shift in research priorities reflects a clear transition from fundamental molecular studies (e.g., “lipid rafts” and “signal transduction”) to disease-specific applications (e.g., “Hippo pathway” and “therapy”), demonstrating a strong trend toward translational and clinical research. Keyword clustering analysis further reinforces these insights, with “Hippo pathway,” “YAP/TAZ,” and “signal transduction” forming a cluster around oncogenic signaling, while “lipid rafts” and “plasma membrane” highlight the importance of membrane dynamics in palmitoylation regulation. Additionally, “mutations” and “overexpression” point to palmitoylation’s role in tumorigenesis, whereas “therapy” and “immunotherapy” suggest its growing relevance in targeted cancer treatments. The presence of keywords like “antiviral activity” and “prognosis” further indicates that palmitoylation research extends beyond oncology into broader biomedical fields. In conclusion, the evolution of keyword trends and clustering patterns illustrates a shift from mechanistic studies to translational applications, with increasing emphasis on oncogenic signaling, immunotherapy, and targeted therapeutic strategies, reinforcing the emerging significance of palmitoylation in oncology and precision medicine.

## 4 Discussion

### 4.1 Bibliometrics research

As research on protein palmitoylation in cancer continues to advance, the volume of studies in this field has grown significantly, reflecting its emerging importance in tumor biology and therapy. However, despite the increasing understanding of its mechanisms and therapeutic potential, a comprehensive bibliometric analysis of the field’s global trends, key research hotspots, and future directions remains lacking. To our knowledge, this is the first bibliometric study to quantify palmitoylation’s evolving role in cancer. Unlike prior reviews focused on molecular mechanisms, our analysis reveals unmet needs, first for limited clinical trials targeting palmitoylation, second for geographic imbalances in translational output, and finally for understudied crosstalk between palmitoylation and epigenetic regulation.​

Between 2004 and 2024, the scientific interest in protein palmitoylation and its role in cancer has grown steadily, with a notable surge in research output post-2020, marking the field’s transition into a rapidly expanding phase. The research focus has evolved, shifting from early investigations into fundamental molecular mechanisms such as “lipid rafts” and “signal transduction” (2004-2016) to cancer-specific mechanisms and clinical applications, including “Hippo pathway” and “immunotherapy” (2016-2024). Keyword co-occurrence analysis revealed three conceptual pillars: palmitoylation as a metabolic regulator, its role in immune checkpoint stabilization, and its crosstalk with oncogenic pathways such as Hippo-YAP/TAZ. These themes align with recent paradigm shifts in cancer biology, where post-translational modifications are increasingly recognized as integrators of metabolic and immune signaling. Notably, the burst keyword “therapy” resonates with preclinical studies targeting ZDHHC enzymes, suggesting a transition from mechanistic exploration towards therapeutic innovation.

Through an analysis of geographic and institutional contributions, it is evident that the United States and China are the dominant contributors, with the U.S. leading in citation impact, indicating a stronger influence on the field’s academic discourse. Institutions such as Harvard University and the Chinese Academy of Sciences are among the top research centers investigating the role of palmitoylation in cancer biology. The U.S. maintains its leadership in this field due to multiple factors, including substantial research funding, top-tier research institutions, and strong interdisciplinary collaboration. Furthermore, the USA attracts top global researchers, benefiting from open immigration policies, high-quality academic environments, and a well-integrated translational research infrastructure. The National Cancer Institute and other funding agencies have also played a critical role in supporting long-term research on cancer signaling and palmitoylation-related mechanisms, further cementing the country’s dominant position in this domain. Additionally, the USA excels in research translation, with strong academic-industry collaborations accelerating the transition from basic research to clinical applications, ensuring its continued leadership in palmitoylation research.

Regarding scientific publication trends, this study found that the Journal of Biological Chemistry is the leading journal in this field, both in terms of publication volume and citation impact, reflecting its central role in disseminating findings on molecular mechanisms, signaling pathways, and protein modifications. Open-access journals such as Nature Communications and PLOS ONE have also played a significant role in the widespread dissemination of palmitoylation research. Highly cited journals like Proceedings of the National Academy of Sciences (PNAS) and Nature further indicate the broad interdisciplinary impact of palmitoylation research, particularly in cellular signaling, tumor progression, and metastasis. Additionally, the high citation frequency of Cancer Research and Cell demonstrates the increasing recognition of palmitoylation’s role in tumor immunology and drug resistance. These findings highlight that palmitoylation research is not only gaining traction in fundamental molecular biology but is also establishing a strong presence in translational oncology and clinical studies, providing a solid platform for future breakthroughs.

In terms of scientific advancements, early studies focused on elucidating the basic mechanisms of palmitoylation and its effects on protein function. However, as its relevance to cancer became clearer, research priorities shifted toward its involvement in tumor metabolism, immune evasion, and potential therapeutic applications. A striking example is the discovery that palmitoylation of PD-L1 plays a critical role in immune evasion in breast cancer, with studies showing that blocking PD-L1 palmitoylation significantly enhances T-cell responses against tumors. Author co-occurrence analysis highlights that leading researchers such as Wang Y and Tate EW have made major contributions to understanding palmitoylation’s role in oncogenic signaling and immune modulation. Similarly, co-citation analysis underscores the influence of key journals like the Journal of Biological Chemistry and Nature Communications, reinforcing their importance in shaping the scientific discourse on palmitoylation and cancer.

Despite the growing significance of palmitoylation in cancer research, several challenges remain. The dynamic regulatory mechanisms of palmitoylation remain incompletely understood, particularly regarding its role in different cancer types and its interactions with other post-translational modifications. Additionally, developing therapeutic strategies targeting palmitoylation remains a major hurdle, as current approaches suffer from low selectivity, poor bioavailability, and off-target effects. Further cancer-specific studies are needed to uncover the unique roles of palmitoylation across different tumor types, emphasizing the necessity for systematic investigations tailored to specific cancer contexts. Moving forward, a deeper exploration of palmitoylation’s mechanistic landscape and therapeutic potential will be essential for advancing precision medicine and developing novel cancer treatment strategies.

### 4.2 Palmitoylation and cancer immune evasion mechanisms

Protein palmitoylation, a reversible protein lipid modification, modulates cancer immune evasion through multi-dimensional mechanisms. Regarding immune checkpoints, DHHC9-mediated palmitoylation of PD-1 enhances its membrane stability, consequently suppressing T-cell function via the Rab11/mTOR pathway. Conversely, DHHC3-catalyzed PD-L1 palmitoylation impedes its ubiquitination-mediated degradation, and 2-bromopalmitate can restore anti-tumor immunity by inhibiting DHHC3 (24, 25). In terms of inflammatory regulation, ZDHHC5 and ZDHHC12 exhibit dual regulatory roles by mediating the palmitoylation-dependent activation or degradation of the inflammasome, specifically NLRP3, respectively (26–28). Within the cGAS-STING pathway, ZDHHC18 inhibits the DNA-binding capability of cGAS, while STING palmitoylation, dependent on VDAC2, promotes interferon secretion; 2-bromopalmitate can block this process (29). Metabolically, palmitate supplied by FASN inhibits hepatocellular carcinoma (HCC) metastasis through CD44 lipid raft sequestration (30).

Conversely, PRMT1 promotes chemoresistance by mediating PHGDH/FASN palmitoylation (31). Therapeutically, while 2-bromopalmitate faces limitations due to toxicity, specific inhibitors targeting DHHC3/DHHC9 or combination therapies with immune checkpoint inhibitors hold greater promise (32). Furthermore, palmitoylation indirectly contributes to immune evasion by modulating T-cell activation proteins such as Lck/LAT (33). Collectively, palmitoylation facilitates tumor immune evasion by integrating immune checkpoint stability, inflammatory microenvironment regulation, and metabolic reprogramming. Therefore, targeting key ZDHHC enzymes or developing precise inhibitors represents a crucial future direction.

### 4.3 Palmitoylation and tumor metabolic reprogramming

As a dynamic and reversible post-translational modification, palmitoylation orchestrates tumor metabolic reprogramming through multifaceted mechanisms. At the signaling pathway level, palmitoylation of key proteins such as AKT, Ras, and PD-1 drives aberrant lipid and energy metabolism, contributing to tumor progression (34).

Direct modification of metabolic enzymes plays a crucial role. S-palmitoylation of PHGDH and FASN promotes chemoresistance in breast cancer, while palmitoylation of ACACA and GPX4 influences lipid metabolic reprogramming and ferroptosis resistance, respectively (35, 36). Within the broader lipid metabolism network, ZDHHC4/5-mediated CD36 membrane localization enhances fatty acid uptake. Furthermore, lipid accumulation in the tumor microenvironment suppresses T-cell function via palmitoylation of PD-L1, and the activity of FASN/SCD is intricately linked to membrane lipid synthesis and signal transduction (37–39).

At the epigenetic level, palmitoylation may indirectly reshape metabolic phenotypes by dynamically regulating histone modifications and the function of RNA m6A-associated proteins through metabolite availability (40, 41).In terms of therapeutic strategies, targeting the ZDHHC family and developing specific drugs, potentially combined with dietary interventions, show promise. However, the toxicity limitations of the broad-spectrum inhibitor 2-BP must be overcome (33).

In summary, palmitoylation drives tumor metabolic reprogramming by integrating signal transduction, metabolic enzyme activity, lipid networks, and epigenetic regulation. Future efforts should therefore prioritize the elucidation of enzyme-substrate specificity and the development of highly selective inhibitors to harness this regulatory network for therapeutic benefit.

### 4.4 Palmitoylation and cancer therapy

With the increasing understanding of palmitoylation mechanisms, targeting palmitoylation has gradually emerged as a promising new approach in cancer therapy. Although clinical applications in this field are still in their early stages, several studies have suggested that targeting depalmitoylases or palmitoyltransferases may provide an effective strategy for cancer treatment (42).

Inhibition of the depalmitoylase ABHD17 has been shown to alter tumor cell proliferation and migration. By modulating the palmitoylation status of proteins on the tumor cell membrane, it is possible to influence the signaling pathways within the tumor cells, thereby inhibiting tumor growth and metastasis. Similarly, members of the ZDHHC family of palmitoyltransferases have been found to play significant roles in various cancer types, making the development of inhibitors targeting ZDHHC a potential new class of anti-cancer drugs (43–45).

However, despite the potential of targeting palmitoylation as a therapeutic strategy, several challenges remain (16). First, selectively modulating palmitoylation without affecting the function of normal cells is a critical issue. Additionally, the stability and selectivity of drugs targeting palmitoylation are key factors influencing their clinical application. Therefore, future research should focus on identifying more specific inhibitors and validating their safety and efficacy through animal models (46).

### 4.5 Palmitoylation and cancer epigenetics

Epigenetic alterations play a critical role in cancer initiation and progression, influencing gene expression patterns and chromatin dynamics without altering the DNA sequence. Emerging evidence suggests that protein palmitoylation, as a post-translational modification, is closely linked to epigenetic regulation in cancer. Beyond its role in modulating protein stability and localization, palmitoylation may also impact gene regulation by altering transcription factor activity and chromatin structure, thereby shaping cancer cell epigenetic landscapes (47).

For instance, palmitoylation has been shown to influence transcription factor function by modifying their nuclear localization and activity. Studies have demonstrated that key oncogenic transcription factors, such as YAP and TAZ, undergo palmitoylation-dependent nuclear trafficking, directly impacting the transcription of tumor-related genes (48). This suggests that palmitoylation serves as a molecular bridge between intracellular signaling pathways and epigenetic regulation, integrating extracellular signals with gene expression control. Such findings underscore the potential role of palmitoylation as an epigenetic regulator in tumorigenesis, offering a novel perspective for exploring cancer epigenetics and developing targeted epigenetic therapies (49).

Additionally, palmitoylation has been implicated in the regulation of chromatin-modifying enzymes. For example, histone deacetylases (HDACs) and methyltransferases can be regulated by palmitoylation, influencing their activity and localization within the nucleus, which in turn modulates chromatin structure and gene expression. This highlights the intricate interplay between protein palmitoylation and chromatin dynamics, suggesting a potential avenue for therapeutic intervention in epigenetic-driven cancer pathways (50).

### 4.6 Limitations

This study has several acknowledged limitations that should be considered when interpreting the findings. First, the dataset was exclusively sourced from the Web of Science Core Collection (WoSCC) for ease of bibliometric analysis, which means that relevant studies indexed in other major academic databases, such as PubMed, Google Scholar, and Embase, were not included, potentially leading to data omission. Second, recently published high-quality studies may have lower citation counts, meaning their impact might be underrepresented in the analysis, affecting the identification of emerging research trends. Third, this study only included English-language publications, which may introduce a language bias and result in the exclusion of relevant non-English research contributions. These limitations should be considered when interpreting the study’s results. However, despite these constraints, this study provides a valuable overview of the research landscape, key focus areas, and emerging trends in palmitoylation and cancer, offering a useful reference for researchers in the field.

## 5 Conclusion

Future efforts should concentrate on three primary directions: constructing high-resolution pan-cancer palmitoylation site maps to unveil subtype-specific modification patterns; developing subtype-selective inhibitors targeting the ZDHHC enzyme family to overcome the toxicity limitations of current broad-spectrum inhibitors; and establishing international research alliances to integrate China’s high productivity with the United States’ translational expertise, thereby bridging regional disparities between basic research and clinical innovation, ultimately advancing palmitoylation regulatory networks toward precision therapeutic strategies.

## Data Availability

The original contributions presented in the study are included in the article/**Supplementary Material**. Further inquiries can be directed to the corresponding author.
