# Supplementary material for: Research trends of protein palmitoylation in cancer from 2004 to 2024: a bibliometric and visualization analysis
Source: Front Oncol. 2025 Jun 23;15:1571870. doi: 10.3389/fonc.2025.1571870 (PMC12229854; doi:10.3389/fonc.2025.1571870)
Supplement: Supplementary file 1 [file DataSheet1.pdf]

Entry Terms:

- Tumors
- Neoplasia
- Neoplasias
- Neoplasm
- Tumor
- Cancer
- Cancers
- Malignant Neoplasm
- Malignancy
- Malignancies
- Malignant Neoplasms
- Neoplasm, Malignant
- Neoplasms, Malignant
- Benign Neoplasms
- Neoplasms, Benign
- Neoplasm, Benign
- Benign Neoplasm

Entry Terms:

- palmitoylation
  protein palmitoylation
- S-palmitoylation

Web of Science Search Strategy (v0.1)

Database: Web of Science Core Collection

Entitlements:

- WOS.IC: 1993 to 2025
- WOS.CCR: 1985 to 2025
- WOS.SCI: 1900 to 2025
- WOS.AHCI: 1975 to 2025
- WOS.ESCI: 2020 to 2025
- WOS.ISTP: 1996 to 2025
- WOS.SSCI: 1900 to 2025
- WOS.ISSHP: 1996 to 2025

Searches:

1: ((((((((((((((TS=(Tumors)) OR TS=(Neoplasia)) OR TS=(Neoplasias)) OR TS=(Neoplasm)) OR TS=(Tumor)) OR TS=(Cancer)) OR TS=(Cancers)) OR TS=(Malignant Neoplasm)) OR TS=(Malignancy)) OR TS=(Malignancies)) OR TS=(Malignant Neoplasms)) OR TS=(Neoplasm, Malignant)) OR TS=(Neoplasms, Malignant)) OR TS=(Benign Neoplasms)) OR TS=(Neoplasms, Benign)) OR TS=(Neoplasm, Benign)) OR TS=(Benign Neoplasm) Editions: WOS.SCI Date Run: Mon May 19 2025 22:00:44 GMT+0800 (中国标准时间) Results: 4652953

2: ((TS=(Palmitoylation)) OR TS=(protein palmitoylation)) OR TS=(S-palmitoylation) Editions: WOS.SCI Date Run: Mon May 19 2025 22:01:09 GMT+0800 (中国标准时间) Results: 5202

3: #2 AND #1 Editions: WOS.SCI Date Run: Mon May 19 2025 22:01:17 GMT+0800 (中国标准时间) Results: 748

4: #2 AND #1 and 2025 or 1977 or 1990 or 1997 or 1998 or 1999 or 2000 or 2001 or 2002 or 2003 (Exclude – Publication Years) Editions: WOS.SCI Date Run: Mon May 19 2025 22:03:00 GMT+0800 (中国标准时间) Results: 705

5: #2 AND #1 and 2025 or 1977 or 1990 or 1997 or 1998 or 1999 or 2000 or 2001 or 2002 or 2003 (Exclude – Publication Years) and Article or Review Article (Document Types) Editions: WOS.SCI Date Run: Mon May 19 2025 22:03:17 GMT+0800 (中国标准时间) Results: 700

6: #2 AND #1 and 2025 or 1977 or 1990 or 1997 or 1998 or 1999 or 2000 or 2001 or 2002 or 2003 (Exclude – Publication Years) and Article or Review Article (Document Types) and Book Chapters or Proceeding Paper or Early Access (Exclude – Document Types) Editions: WOS.SCI Date Run: Mon May 19 2025 22:03:23 GMT+0800 (中国标准时间) Results: 693

7: #2 AND #1 and 2025 or 1977 or 1990 or 1997 or 1998 or 1999 or 2000 or 2001 or 2002 or 2003 (Exclude – Publication Years) and Article or Review Article (Document Types) and Book Chapters or Proceeding Paper or Early Access (Exclude – Document Types) and Retracted Publication (Exclude – Document Types) Editions: WOS.SCI Date Run: Mon May 19 2025 22:03:28 GMT+0800 (中国标准时间) Results: 689

8: #2 AND #1 and 2025 or 1977 or 1990 or 1997 or 1998 or 1999 or 2000 or 2001 or 2002 or 2003 (Exclude – Publication Years) and Article or Review Article (Document Types) and Book Chapters or Proceeding Paper or Early Access (Exclude – Document Types) and Retracted Publication (Exclude – Document Types) and Chinese or French (Exclude – Languages) Editions: WOS.SCI Date Run: Mon May 19 2025 22:03:59 GMT+0800 (中国标准时间) Results: 668
